# Cost of wastewater-based environmental surveillance for SARS-CoV-2: Evidence from pilot sites in Blantyre, Malawi and Kathmandu, Nepal

**DOI:** 10.1371/journal.pgph.0001377

**Published:** 2022-12-27

**Authors:** Lucky G. Ngwira, Bhawana Sharma, Kabita Bade Shrestha, Sushil Dahal, Reshma Tuladhar, Gerald Manthalu, Ben Chilima, Allone Ganizani, Jonathan Rigby, Oscar Kanjerwa, Kayla Barnes, Catherine Anscombe, Joseph Mfutso-Bengo, Nicholas Feasey, Mercy Mvundura

**Affiliations:** 1 Malawi Liverpool Wellcome Research Programme, Blantyre, Malawi; 2 Department of Clinical Sciences, Liverpool School of Tropical Medicine, Liverpool, United Kingdom; 3 Environment and Public Health Organization, Kathmandu, Nepal; 4 Innovative Solution Pvt. Ltd, Lalitpur, Nepal; 5 Department of Microbiology, Tribhuvan University, Kathmandu, Nepal; 6 Department of Planning and Policy, Ministry of Health, Lilongwe, Malawi; 7 Public Health Institute of Malawi, Ministry of Health, Lilongwe, Malawi; 8 Environmental Department, Ministry of Health, Lilongwe, Malawi; 9 Institute of Infection, Veterinary and Ecological Sciences, University of Liverpool, Liverpool, United Kingdom; 10 Harvard School of Public Health, Boston, Massachusetts, United States of America; 11 Broad Institute of MIT and Harvard, Cambridge, Massachusetts, United States of America; 12 University of Glasgow MRC Centre for Virus Research, Glasgow, United Kingdom; 13 Kamuzu University of Health Sciences, Blantyre, Malawi; 14 Medical Devices and Health Technologies, PATH, Seattle, Washington, United States of America; University of Oslo Faculty of Medicine: Universitetet i Oslo Det medisinske fakultet, NORWAY

## Abstract

Environmental surveillance of rivers and wastewater for SARS-CoV-2 detection has been explored as an innovative way to surveil the pandemic. This study estimated the economic costs of conducting wastewater-based environmental surveillance for SARS-CoV-2 to inform decision making if countries consider continuing these efforts. We estimated the cost of two SARS-CoV-2 environmental surveillance pilot studies conducted in Blantyre, Malawi, and Kathmandu, Nepal. The cost estimation accounted for the consumables, equipment, and human resource time costs used for environmental surveillance from sample selection until pathogen detection and overhead costs for the projects. Costs are reported in 2021 US$ and reported as costs per month, per sample and person per year. The estimated costs for environmental surveillance range from $6,175 to $8,272 per month (Blantyre site) and $16,756 to $30,050 (Kathmandu site). The number of samples processed per month ranged from 84 to 336 at the Blantyre site and 96 to 250 at the Kathmandu site. Consumables costs are variable costs influenced by the number of samples processed and are a large share of the monthly costs for ES (ranging from 39% to 72%). The relatively higher costs per month for the Kathmandu site were attributable to the higher allocation of dedicated human resources and equipment to environmental surveillance for SARS-CoV-2 compared to the Blantyre site where these resources were shared with other activities. The average cost per sample ranged from $25 to $74 (Blantyre) and $120 to $175 (Kathmandu). There were associated economies of scale for human resources and equipment costs with increased sample processing and sharing of resources with other activities. The cost per person in the catchment area per year ranged from $0.07 to $0.10 in Blantyre and $0.07 to $0.13 in Kathmandu. Environmental surveillance may be a low-cost early warning signal for SARS-CoV-2 that can complement other SARS-CoV2 monitoring efforts.

## Introduction

Low- and middle-income countries (LMICs) face the twin challenges of high infectious disease burden and small health budgets. The existing strain on resources in LMICs has been exacerbated by outbreaks of the severe acute respiratory syndrome coronavirus (SARS-CoV-2), which causes coronavirus disease-19 (COVID-19) [1]. To curb the spread of SARS-CoV-2, governments around the world have implemented diverse public health interventions [2], e.g., partial or complete lockdowns, mask distribution, and mask-wearing mandates. These interventions have had substantial economic implications and have been reactive rather than proactive [3].

Epidemiological surveillance based on testing of symptomatic individuals has been used in many countries to provide a warning signal that public health authorities can use to inform decisions about implementing measures to flatten the epidemic curve [4, 5]. However, screening of symptomatic individuals in the community has been particularly challenging in low-income settings. As such, waves of SARS-CoV-2 infections have often only become apparent posteriori, through testing of people receiving care at medical facilities, and thus well after the wave has become established [6, 7]. As the world continues to grapple with waves of SARS-CoV-2 infections, often due to the emergence of new variants of concern, it has become clear that innovative and lower-cost surveillance solutions must be explored to identify future waves of SARS-CoV-2 infections in a timely manner. These must provide an early warning system to give public health authorities time to implement interventions to reduce the intensity and magnitude of waves of SARS-CoV-2 infections.

Environmental surveillance has been used for epidemiological surveillance of pathogens in the past and has been of value in the polio eradication endgame [4]. More recently, ES has been used as a tool for surveillance of COVID-19, and there is evidence showing that SARS-CoV-2 can be detected from sewer and wastewater samples prior to observed clinical peaks [8–13].

While not replacing clinical testing, wastewater ES for SARS-CoV-2 is expected to be cheaper and more logistically feasible than surveillance through mass population screening [14]. Although LMICs could benefit from implementing ES as a potentially low-cost surveillance strategy for SARS-CoV-2, it represents a new technology with cost implications. To date, only two studies have assessed the costs and cost-effectiveness of ES as an intervention for SARS-CoV-2. However, both studies have been conducted in high-income countries and therefore, there is no existing evidence from LMIC [7, 15]. This paper reports on the cost of ES for SARS-CoV-2 as an add-on tool for continued SARS-CoV-2 monitoring in Malawi and Nepal. Evidence on the cost associated with ES in LMIC settings is needed to inform policymakers, donors, and other stakeholders to support the planning and budgeting for resource allocation on strategies to monitor the ongoing SARS-CoV-2 pandemic.

## Material and methods

### Overview of the process for ES for SARS-CoV-2

ES for SARS-CoV-2 involves four main steps i) sample collection is done from wastewater (e.g., from sewers, wastewater treatment plants, or rivers), ii) these samples are then concentrated in the laboratory followed by, iii) RNA extraction, and iv) pathogen detection using quantitative real-time polymerase-chain-reaction (PCR), also done in the laboratory. The context for conducting ES for SARS-CoV-2 are described briefly below for each pilot site included in this evaluation.

### Pilot sites and site-specific methods for ES for SARS-CoV-2

#### Blantyre, Malawi pilot site

Blantyre is largely not served by a formal, centralised wastewater network, instead, most waste material and sewage are deposited and transported via an urban freshwater river system that is contaminated directly or through emptying of the contents of pit latrines. The Blantyre pilot site had previously been conducting ES activities for other pathogens and hence the sample collection sites that had been identified for typhoid ES had SARS-CoV-2 surveillance added on. From May 1 to December 31, 2020, wastewater samples were collected daily from one sewer and six river sites running through three high-density dwelling locations of Blantyre city (population of about 1 million people) [16]. From January 1, 2021 to December 31, 2021, the number of sample collection sites expanded from 7 to 80, with sample collection occurring every fortnight from each site, and these sites have previously been described [17]. Of note, by expanding to these sites 80, the became representative of the population of the entire city, excepting those parts of the city served by septic tanks or sewers. For each site, grab samples were collected by hand for viral analysis and transported to a laboratory facility at the Malawi-Liverpool-Wellcome Research Programme (MLW) within Blantyre. Water samples were then concentrated using 10% polyethylene glycol, which pulls down organic matter in the water sample, including DNA and RNA that can be pelleted and then extracted using any commercially available RNA extraction kit (Qiagen QIAamp Viral RNA Mini Kit). Detection of SARS-CoV-2 was performed using the CDC N1 real-time PCR assay, the same assay used locally to detect SARS-CoV-2 from patient samples [18].

#### Kathmandu, Nepal pilot site

Kathmandu Valley Water Supply Management Board’s responsibility for the overall management of wastewater is limited to the Kathmandu, Patan Bhaktapur valley only and there are insufficient numbers of wastewater treatment plant that are functional. Wastewater from remaining municipalities is directly discharged into Bagamati River and its tributaries. The faecal sludge from onsite sanitation systems in the valley is emptied by the private desludging service provider which is dumped into the open environment without any treatment. At the Kathmandu pilot site, ES was a new activity which was started in March 2021. From March to June 2021, wastewater samples were collected twice a month, on every alternate week, from sewers and river sites running through Kathmandu Valley, Nepal, a city with a population size of about 2.7 million people. From July 2021 to December 2021, wastewater grab samples were collected from the sewers and river sites every week. Altogether, samples were collected from 23 sampling points categorized as municipal sewers at SARS-CoV-2 hotspots, squatter areas, sewers from housing (apartment), inlets of the wastewater treatment plant and river sites, and a SARS-COV-2 hospital sewer. Grab samples from study areas were collected for the ES activity for both SARS-CoV-2 detection as well as physicochemical analysis. The pH and temperature of all samples were analyzed on-site with the help of a field pH meter (Lutron PH-222). Sample processing and analysis were conducted in the Central Department of Microbiology Laboratory at Tribhuvan University, Kirtipur, Kathmandu, Nepal. Collected water samples were then concentrated using the skimmed milk flocculation method; nucleic acids were then extracted using the Qiagen QIAamp Viral RNA mini kit. Detection of SARS-CoV-2 was performed using the N1 and E1 real-time PCR assay [11].

### Costing methods

The study used a micro-costing approach to estimate the economic costs per month, per sample and per person per year for ES for SARS-CoV-2 from the health system perspective. The costing data collection and analysis were done using a Microsoft Excel-based costing template. Staff working at the two SARS-CoV-2 ES study sites in Blantyre, Malawi, and Kathmandu, Nepal, were interviewed to collect the data for the activities for which they were responsible, and the associated resources used for each ES activity. The ES activities included in the costing are the four steps as outlined above (i.e., sample collection, concentration, RNA extraction, and pathogen detection). Secondary data on unit prices, such as salaries, consumables, and equipment, were obtained from the financial records of the projects for each study site.

The micro-costing approach used an ingredient-based method to account for the resources used in each ES activity. Financial costs that included direct monetary outlays were included in the costing, and so were the opportunity costs for using existing resources [19], the aggregation of financial and opportunity costs providing the economic cost estimates. The resources included in the costing were consumables, labor, transport, equipment, and overhead. Costs for consumables were estimated based on the number used per sample and these quantities were then multiplied by the respective unit cost (Table 1). Consumable costs per month varied based on the number of samples processed per month. For labor or human resources, the monthly salary and benefits for each staff involved in ES for SARS-CoV-2 were included (Table 2). When staff were involved in another activity, an allocation was made to ES for SARS-CoV-2 based on the percentage of time they spent on the activity. The human resource costs were not varied by the number of samples processed but accounted for through the monthly labor costs. Sample collection incurred a transport cost; both sites used hired vehicles to transport the teams to the sampling sites and this expenditure for the vehicles was included. For equipment, a useful life was assumed, and replacement costs for the equipment were annualized using a 3% discount rate. Where equipment was shared with other activities, the allocation to ES for SARS-CoV-2 was done based on utilization rates. Table 3 shows the procurement prices for equipment and allocations for SARS-CoV-2 ES. Overhead costs were included based on actual expenditure allocated to ES for SARS-CoV-2.

**Table 1:** Unit prices for consumables and reusable supplies in 2021 US$.

		Blantyre site	Kathmandu site
	Unit of measure	Unit price	Unit price
*Consumables*:			
Alcohol wipe	each	$0.05	NA
CDC diagnostic kit (IDT)–official & research	per sample	$0.67	N/A
Qscript one step	ml	$2.08	N/A
Qiagen QIAamp Viral RNA Mini Kit	per kit	NA	$54.79
Nuclease free water	ml	$5.69	$4.64
Parafilm x1 length	pack	$82.00	$47.52
Eppendorf 1.5ml	each	$0.06	N/A
Eppendorf 2ml	each	$0.05	N/A
Ethanol (70%)	ml	$0.16	$0.008
DNA low binding tube (1.5 ml)	each	NA	$0.17
Falcon conical tube	each	$1.03	$0.50
Dropper	each	N/A	$0.07
Microfuge tube	each	N/A	$0.03
Face shield	each	N/A	$0.75
Gloves	pair	$0.14	$0.14
GoTaq Wastewater SARS-CoV2 RTq PCR system	220 ml	N/A	$23.66
HCI concentrate	500ml	N/A	$3.47
Mask-surgical	each	$0.09	N/A
Mask-N95	each	$1.27	$0.91
PCR Strips	each strip	N/A	$0.62
Pipette tip P1000 μL	each	$0.08	$0.01
Pipette tip P200 μL	each	$0.08	$0.02
(PBS) for PCR	ml	$0.01	$0.04
PCR plate	each	$3.54	N/A
Plate seal	each	$1.16	N/A
Plastic apron/coat	each	$0.29	N/A
Polyethylene Glycol (PEG) for concentration	ml	$0.41	N/A
Qiagen Bviral mini kit	each	$8.30	N/A
Rnase/Dnase free water	ml	$0.62	N/A
Skim milk powder	g	N/A	$22.31
Sodium Chloride (Na Cl)	mL	$0.38	N/A
Sodium Hydroxide (NaoH)	g	N/A	$4.08
Tissue paper (paper towel)	per roll	$0.41	$0.25
Kim wipes for nanodrop	per sheet	N/A	$0.07
*Reusable supplies*:			
Sample collection bottle 125 ml	each	N/A	$1.11
Sample collection bottle 500 ml	each	$12.65	$2.02
Serosurgical pipettes 25 ml	each	N/A	$2.87
Serosurgical pipettes 10 ml	each	N/A	$2.59
Sterile 1 liter sample bottle	each	$22.77	N/A
Dispenser/wash 100ml bottle manual	each	$25.00	N/A
PPE suit	each	N/A	$24.49

*N/A not applicable.

**Table 2:** Monthly salary and benefits for key personnel involved in ES activities in 2021 US$ and allocation of monthly salary to ES for SARS-CoV-2.

	Monthly salary and benefits	Allocation of monthly salary to ES for SARS-CoV-2
*Blantyre site*:		
Field team leader	$756	50%
Field workers	$526 to 589	50%
Laboratory Scientist	$790	10%
*Kathmandu site*:		
Technical person	$450	100%
Research assistant	$215 to $239	100%
Research associate	$286	100%

**Table 3:** Procurement prices for equipment in 2021 US$, allocation to ES for SARS-CoV-2, and assumed useful life.

	Blantyre site		Kathmandu site		Assumed useful life (years)
	Full procurement price (US$)	Allocation to ES for SARS-CoV-2	Full procurement price	Allocation to ES for SARS-CoV-2	Both countries
*Capital equipment*:					
Quantitative PCR (RT-qPCR)	$25,641	10%	$24,653	100%	10
PCR cabinet	N/A*	N/A	$2,200	100%	10
PCR hood	$12,851	10%	N/A		10
PCR plate centrifuge/ mini spinner	$1,282	10%	$141	100%	10
Vortex	$1,536	10%	$176	100%	10
Microcentrifuge for RNA extraction	$769	10%	$4,150	50%	10
Pipet-aid (Drummond or equivalent)	N/A	N/A	$664	100%	10
Large centrifuge	$9,984	10%	$10,102	50%	10
Electronic weighing balance	$1,285	10%	$840	100%	10
UPS for sequencers and workstation	$2,308	10%	N/A	N/A	10
Autoclave	N/A	N/A	$4,482	50%	10
Freezer –80degrees	$20,513	10%	$2,908	100%	10
Freeze –20degrees (Refrigerator)	$579	10%	$415	100%	10
Coolant box	$52	100%	$25	100%	3
*Other smaller equipment*:					
Alcohol thermometer	$8.22	100%	$2.91	100%	3
Beaker 250mL (sampling cup)	$8.22	100%	$4.15	100%	3
pH temperature meter	$94.88	100%	$320	100%	3

*N/A not applicable.

At the Blantyre pilot site, the number of samples that could be processed varied from a low of 84 samples per month to 168 samples per month (optimal capacity) to a high of 336 samples per month (maximum given the assumed utilization of staff and equipment). At the Kathmandu pilot site, 96 samples were collected per month and the number of samples that could be processed varied from a low of 96 samples per month to 135 samples per month (optimal capacity) to a high of 250 samples per month (maximum given the assumed utilization of staff and equipment).

All the costs are reported in 2021 US dollars (US$). Exchange rates of 790.43 per Malawi Kwacha and 120.47 per Nepalese Rupee were used when converting prices from local currency. The key metrics estimated in this costing study were total cost per month, the cost per sample processed, and the annual cost per person. The cost per month was estimated by adding the monthly resource costs for each of the four activities. The cost per sample was calculated by dividing the total cost per month by the number of samples processed in a month, taking into account the variation in processing capacity that could be achieved. The annual cost per person was calculated by dividing the annual cost by the total catchment population for the geographical area covered by the ES sampling sites—about 1 million in Malawi and approximately 2.7 million in Nepal.

### Ethical review for the costing study

The costing study was determined to not be human studies research by PATH Research Determination Committee and did not need United States ethics committee oversight. The Malawi College of Medicine (now KUHES) Research Ethics Committee exempted the study from further ethical review, as no human samples were involved, but used the existing waiver for the ongoing environmental surveillance (P.07/20/3089). The Nepal Health Research Council (NHRC) exempted the study from further ethical review, as no human samples were involved.

## Results

### Monthly costs

**Fig 1:**
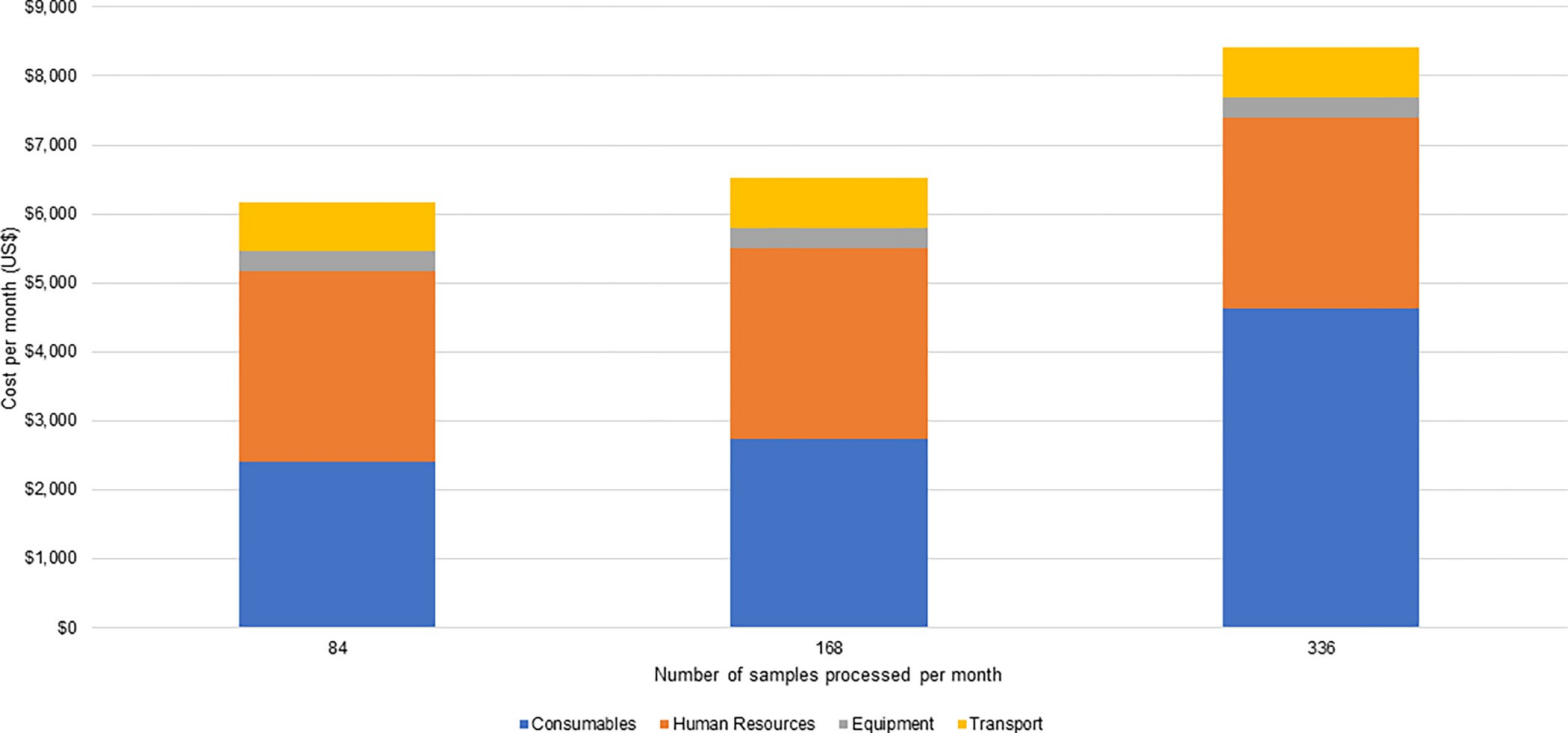
Monthly costs for SARS-CoV-2 ES in 2021 US$ at the Blantyre pilot site.

**Fig 2:**
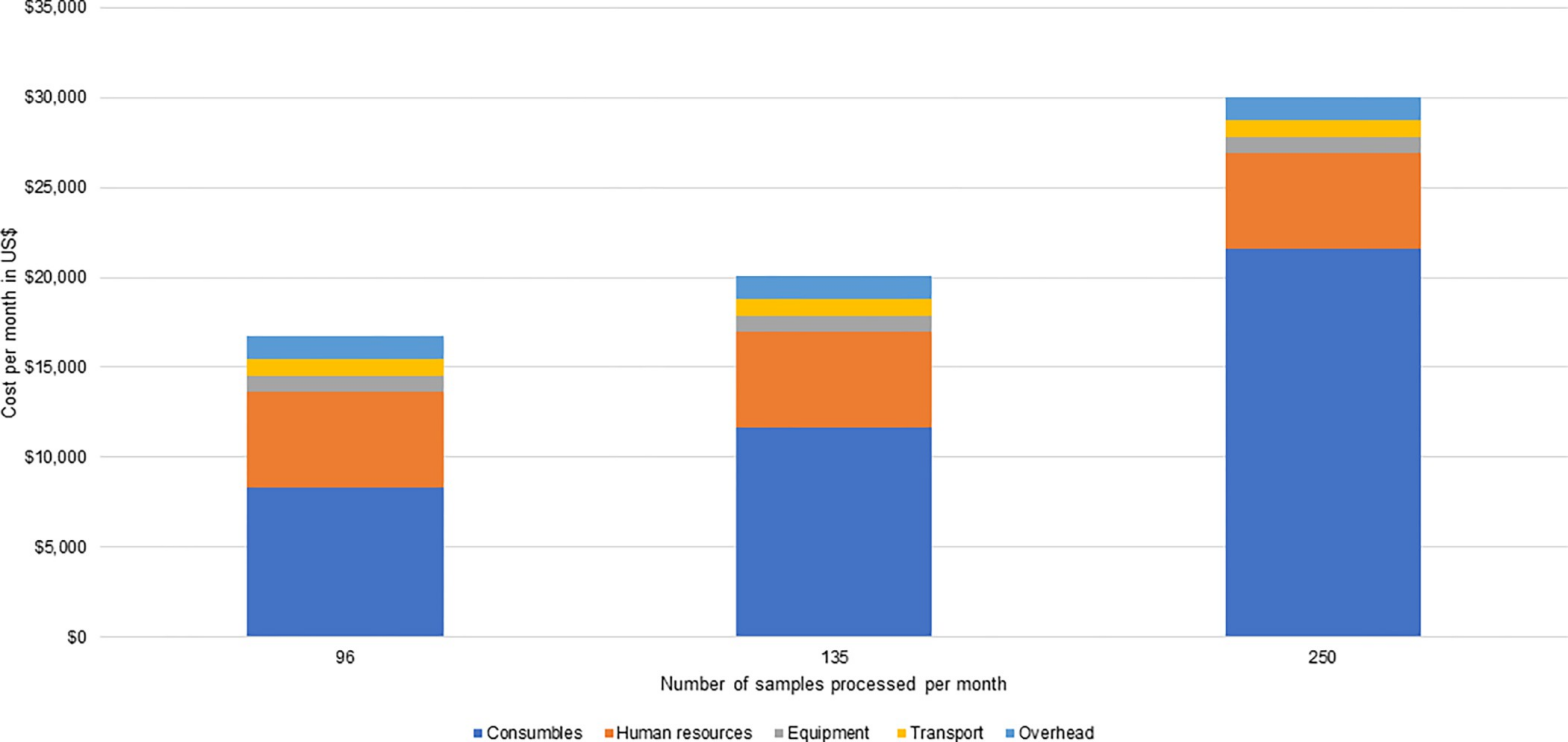
Monthly costs for SARS-CoV-2 ES in 2021 US$ at the Kathmandu pilot site.

The cost of running samples per month is shown in Figs 1 and 2. At the Blantyre pilot site (Fig 1), consumable costs accounted for a significant share of the costs (39% to 71%), and as expected, these costs increased in proportion to the number of samples processed per month. Human resource costs were also a large share of costs (21% to 45%) as some processes are labor-intensive. Still, the proportion of human resource costs declined as the number of samples processed increased due to more efficient utilization of existing human resources. As the number of samples processed increased, there were economies of scale achieved for some resources, such as human resources and equipment, until they reached their total utilization capacity. Equipment costs were a small share of costs, as these resources were shared with other activities and not just for ES for SARS-CoV-2. The total monthly costs for ES at the Blantyre pilot site ranged from $6,175, when 84 samples were processed per month, to $8,146, when 336 samples were processed per month.

Resources also accounted for the largest share of costs at the Kathmandu pilot site (Fig 2): consumables accounted for between 49% and 72% of total costs, depending on the number of samples processed, while human resources accounted for between 18% and 32%, also depending on the number of samples processed. Total costs per month at the Kathmandu pilot site ranged from $16,756 when 96 samples were processed per month, to $30,050, when 250 samples were processed per month. The relatively higher costs per month at the Kathmandu site were attributable to the greater allocation of human resources and equipment to ES for SARS-CoV-2 compared to the Blantyre pilot site, where these resources were shared with other activities.

### Cost per sample

**Fig 3:**
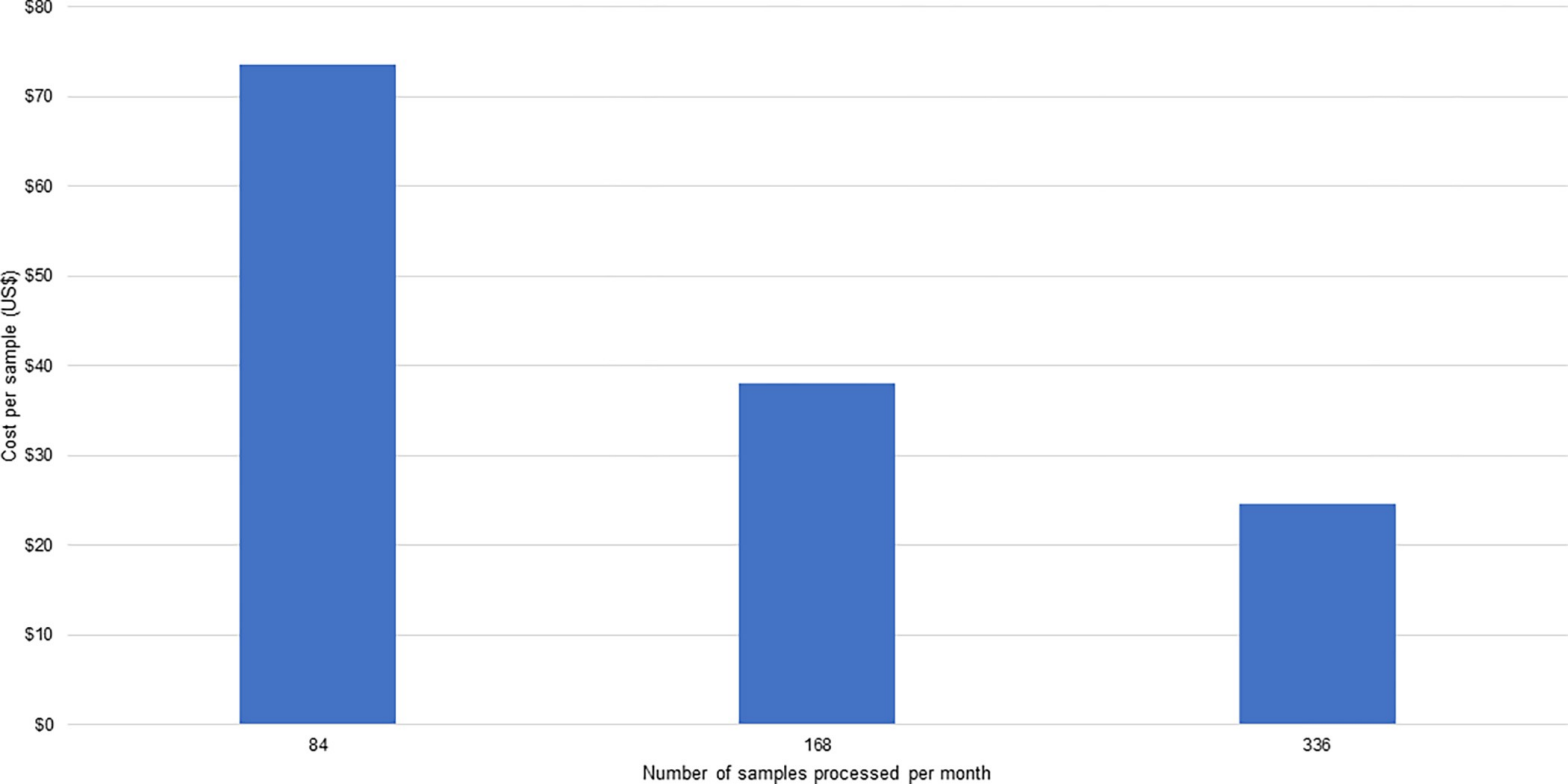
Per sample cost in US$, processed in a month, at the Blantyre pilot site.

**Fig 4:**
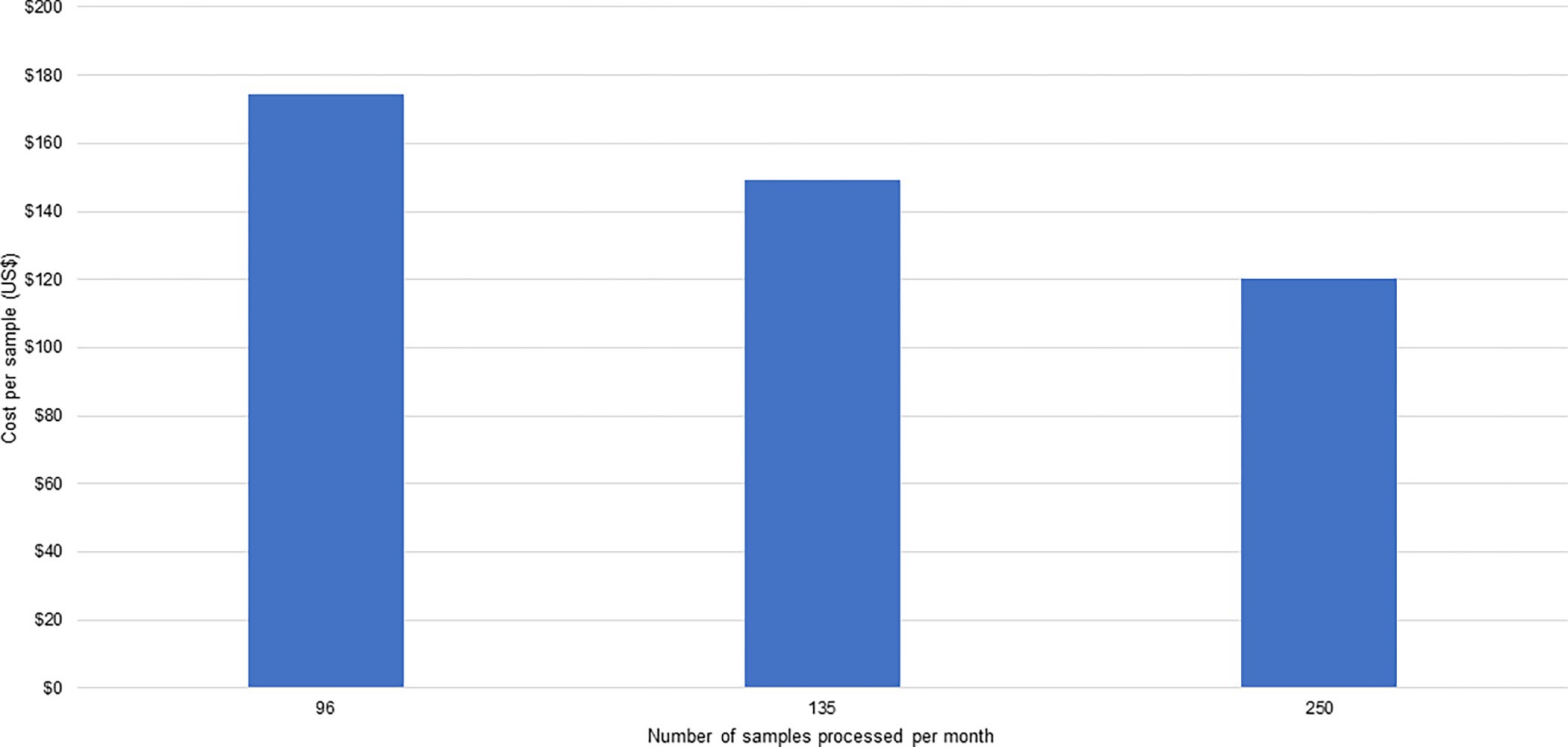
Per sample cost in US$, processed in a month, at the Kathmandu pilot site.

The cost per sample is shown in Figs 3 and 4. For the Blantyre pilot site (Fig 3), the average cost per sample ranges from a high of $74 (when 84 samples were processed per month) to a low of $25 (when 336 samples were run per month). For the Kathmandu pilot (Fig 4), the cost per sample ranges from a high of $175 when 96 samples were processed per month to a low of $120 when 250 samples were processed per month. As noted above, consumable and human resource costs were the most significant components of these cost estimates. The results show efficiency gains from processing a larger number of samples per month.

### Cost per person per year

For the Blantyre pilot, the cost per person per year for ES ranged from $0.07 to $0.10, based on the population for the catchment area in Blantyre. For the Kathmandu pilot, the cost per person per year was very similar and ranged from $0.07 to $0.13, based on the population for the catchment area in Kathmandu Valley.

## Discussion

To our knowledge, this is the first study to report on the cost of ES for SARS-CoV-2 in LMICs and provides evidence of the costs for ES for SARS-CoV-2 in Malawi and Nepal. The main factors that drive variations in cost estimates for ES for SARS-CoV-2 include differences in resourcing and the ability to cost share resources. Unlike the Kathmandu pilot site, where all the laboratory staff and equipment were dedicated to using ES for SARS-CoV-2, at the Blantyre pilot site, staff worked on other activities in addition to ES for SARS-CoV-2 and equipment is used for other ES activities, thereby reducing the per sample cost. These findings from the two pilot sites can provide policymakers and other stakeholders with information to consider for SARS-CoV-2 ES implementation and scale-up.

The cost estimates we present show that consumable costs are the main drivers of the monthly costs of conducting ES for SARS-CoV-2. Therefore once the equipment is procured and the system is established, ongoing costs of procuring consumables and paying human resources, still remain a significant financial outlay that needs planning and budgeting.

Our estimated cost per sample was higher for the Kathmandu pilot site than for the Blantyre pilot site because of the dedication of human resources and the equipment for ES for SARS-CoV-2 alone. However, our cost per person per year estimates from the two pilot sites were in the same range and were also similar to the estimates for rural areas from a costing study done in the USA, which estimated these costs to be approximately $0.10 in rural areas, but much lower in urban areas where they are estimated at approximately $0.005 per person per year [14]. This finding may imply that labor costs, population size and other factors may explain differences in findings when comparing the cost per person per year estimates for USA urban areas to the two pilot sites which were located in major cities of the two study countries. While there has been mixed evidence of SARS-CoV-2 RNA in river water in high-income countries [9, 20], this is different in LMICs where SARS-CoV-2 detection in river water has been observed to be the case [14, 21–24]. ES SARS-CoV-2 may therefore offer a potential low-cost strategy to inform SARS-CoV-2 public health measures in resource-limited- settings [25]. Therefore, ES for SARS-CoV-2 may be a valuable surveillance tool that will complement other interventions to monitor for SARS-CoV-2 in communities.

The evidence of economies of scale implies that for ES to be worth the investment, it will require both the processing of an optimized number of samples and leveraging opportunities for tracking multiple pathogens (i.e., poliovirus or *Salmonella enterica*) and investigating other sample types i.e., food. While ES may not be applicable to other diseases equally across the region, once established the laboratory capacity can be adapted to local public health requirements. Thus, despite the high laboratory set up and unit costs for some capital equipment, the unit costs are lowered when the equipment is used optimally—for multiple diseases and multiple geographies within the same country—and could be leveraged for diagnostic testing of clinical samples.

In this study we compared how the per sample costs change at different levels of sample throughput to evaluate how laboratory staff workload and equipment utilization rates impact the cost estimates. Unlike with clinical diagnostic laboratories, where workload or equipment utilization is dependent on clinical demand, ES workload and equipment utilization can be managed at optimal levels through changing throughput. These data showing how costs vary with throughput can provide evidence for managers on how sample frequency impacts public health data sufficiency and help inform optimal use of resources. Additionally, some efficiencies could be gained by changing the methods used for concentration, which at the Kathmandu pilot site were time-consuming. However, some of these costs we report reflect pilot costs and may not reflect scale up or the most efficient cost scenario.

The study has several limitations. First, the study was done in the context of a pilot setting, and hence costs do not reflect costs associated with the scale-up of ES to other parts of the country in the study countries. In addition, as the costing was done in the context of pilot projects, some of the procurement of supplies and equipment was done internationally for research purposes. If procurement was done through the government sector, the costs may be different. It is likely that the costs presented here are inflated but still indicative to show that ES for SARS-CoV-2 is a low-cost tool that could complement other efforts to monitor for the presence of SARS-CoV-2 infections in communities. Another limitation of the study is that the analysis included costs from one laboratory included in the pilot in each country, so the variations across laboratories could not be captured. We did not evaluate the cost-effectiveness of ES SARS-CoV-2 for SARS-CoV-2 detection in this article, but this evaluation is underway. Lastly, the context of Blantyre and Kathmandu do not present all ES use cases given the value of ES may be more in high density populations that are either served by sewage systems or where there are accessible septic tanks where human waste accumulates and can readily be sampled.

## Conclusion

The cost per person per sample for wastewater-based ES for SARS-CoV-2 in Blantyre and Kathmandu is low, being just a few cents per person per year. This cost per sample is associated with economies of scale with increased sample processing. ES for SARS-CoV-2, therefore, has the potential to be a low-cost surveillance intervention that can complement other interventions and provide early warning signals to decision-makers and thus inform public health measures that flatten waves of SARS-CoV-2 infections.

## Supporting information

S1 File Supplementary tables with types and quantities of consumables used per month for processing environmental surveillance samples at the pilot study site. (DOCX)
