# Supplementary material for: Cost of wastewater-based environmental surveillance for SARS-CoV-2: Evidence from pilot sites in Blantyre, Malawi and Kathmandu, Nepal
Source: PLOS Glob Public Health. 2022 Dec 27;2(12):e0001377. doi: 10.1371/journal.pgph.0001377 (PMC10021894; doi:10.1371/journal.pgph.0001377)
Supplement: S1 File — (DOCX) [file pgph.0001377.s001.docx]

Table A. Types and quantities of consumables used per month for processing 84 samples – Blantyre pilot site.

| **Type of consumable** | **Quantity used per sample month** | **Unit of measure** |
| --- | --- | --- |
| **Activity: Sample collection** |  |  |
| Alcohol wipes | 200 | each |
| Distilled water | 1000 | ml |
| Ethanol (70%) | 350 | mL |
| Gloves | 100 | pairs |
| Masks | 60 | each |
| Parafilm for ceiling sample | 1 | roll |
| Paper towel | 2 | roll |
|  |  |  |
| **Activity: Sample concentration** |  |  |
| Eppendorf 1.5ml | 168 | 1.5 ml bottles |
| Eppendorf 2ml | 168 | 2 ml bottles |
| Falcon 50mL conicle tube | 336 | 1 box |
| Pipette P1000 | 336 | each |
| Pipette P200 | 336 | each |
| PBS for PCR | 336 | ml |
| Polythylene Glycol (PEG) for concentration | 336 | ml |
| Sodium Chloride (Na Cl) | 336 | ml |
|  |  |  |
| **Activity: Pathogen extraction** |  |  |
| Eppendorf 2ml | 336 | 2 ml btls |
| Ethanol (96-100%) | 168 | ml |
| Mask N95 | 7 | each |
| Plastic apron/ coat | 7 | each |
| Pipette P1000µL | 336 | each |
| Pipette P200µL | 336 | each |
| Qiagen Bviral mini kits | 168 | each |
|  |  |  |
| **Activity: Pathogen detection** |  |  |
| CDC diagnostic kit (IDT) -official & research | 96 | 1 kit |
| Covid-19 N gene positive control | 1 | each |
| Eppendorf 2ml | 2 | each |
| Gloves | 4 | boxes |
| PCR plate | 1 | each |
| Plate seal | 1 | each |
| Pipette P20µL | 24 | each |
| Pipette P200µL | 2 | each |
| RNase/Dnase free water | 24 | ml |

Table B. Types and quantities of consumables used per month for processing 96 samples – Kathmandu pilot site

| **Type of consumable** | **Quantity used per sample month** | **Unit of measure** |
| --- | --- | --- |
| **Activity: Sample collection** |  |  |
| Ethanol (70%) | 1840 | ml |
| Gloves | 552 | pairs |
| Mask | 240 | Masks |
| Tissue paper sheets | 80 | each |
|  |  |  |
| **Activity: Sample concentration** |  |  |
| Skim milk powder | 4800 | mg |
| HCl | 96 | ml |
| NaOH | 48 | gm |
| PBS | 288 | ml |
| Serological pipet (25ml) | 96 | each |
| Falcon tubes | 192 | each |
| Micropipette tips (1000 μl) | 384 | each |
| Dropper | 288 | each |
| Microfuge tube 2ml | 288 | each |
|  |  |  |
| **Activity: Pathogen extraction** |  |  |
| GoTaq Wastewater SARS-CoV2 RTq PCR system N1 gene | 3 | kit |
| GoTaq Wastewater SARS-CoV2 RTq PCR system N2 gene | 3 | kit |
| GoTaq Wastewater SARS-CoV2 RTq PCR system E gene | 3 | kit |
| 1.5 ml DNA low binding tube | 192 | each |
| 0.2 ml PCR tubes (strip) | 192 | strips |
| 200μl pipette tip | 192 | each |
| 1μl pipette tip | 192 | each |
| 70% ethanol for surface sterilizing | 192 | ml |
| Nuclease free water | 192 | ul |
|  |  |  |
| **Activity: Pathogen detection** |  |  |
| Qiagen QIAamp Viral RNA Mini Kit | 96 | kit |
| Ethanol (96-100%) | 53.76 | ul |
| Ethanol (70%) | 960 | ml |
| 1.5 ml DNA low binding tube | 288 | each |
| 1000μl pipette tip | 480 | each |
| 200μl pipette tip | 288 | each |
| 1μl pipette tip | 288 | each |
| Kim wipes for nanodrop | 192 | each |
| Parafilm | 0.96 | roll |
|  |  |  |
